# Supplementary material for: Hot spot prediction in protein-protein interactions by an ensemble system
Source: BMC Syst Biol. 2018 Dec 31;12(Suppl 9):132. doi: 10.1186/s12918-018-0665-8 (PMC6311905; doi:10.1186/s12918-018-0665-8)
Supplement: Supplementary file 2 — All relevant MATLAB code. A simple Matlab implement of our predictor. (ZIP 19,742 kb) [file 12918_2018_665_MOESM2_ESM.zip › Additional file 2/Read ASA/ASA Property/SKEMPI/2JELP.txt]

 NetSurfP - Protein Surface Accessibility and Secondary Structure Predictions

  

|  |  |  |
| --- | --- | --- |
|  |  | NetSurfP - Protein Surface Accessibility and Secondary Structure Predictions  Technical University of Denmark |

  


---

```
# For publication of results, please cite:
# A generic method for assignment of reliability scores applied to solvent accessibility predictions.
# Bent Petersen, Thomas Nordahl Petersen, Pernille Andersen, Morten Nielsen and Claus Lundegaard
# BMC Structural Biology 2009, 9:51 doi:10.1186/1472-6807-9-51
#
# Column 1: Class assignment - B for buried or E for Exposed - Threshold: 25% exposure, but not based on RSA
# Column 2: Amino acid
# Column 3: Sequence name
# Column 4: Amino acid number
# Column 5: Relative Surface Accessibility - RSA
# Column 6: Absolute Surface Accessibility
# Column 7: Z-fit score for RSA prediction
# Column 8: Probability for Alpha-Helix
# Column 9: Probability for Beta-strand
# Column 10: Probability for Coil
E M  2JELP                  1    0.639 127.884  -0.703   0.003   0.003   0.994
E F  2JELP                  2    0.420  84.314   0.845   0.004   0.616   0.381
E Q  2JELP                  3    0.543  96.944   0.913   0.001   0.900   0.099
E Q  2JELP                  4    0.273  48.758   1.558   0.001   0.959   0.040
E E  2JELP                  5    0.548  95.823   1.583   0.001   0.959   0.040
B V  2JELP                  6    0.061   9.345   1.120   0.000   0.983   0.017
E T  2JELP                  7    0.404  56.007   1.732   0.000   0.983   0.017
B I  2JELP                  8    0.039   7.234   0.558   0.001   0.959   0.040
E T  2JELP                  9    0.443  61.500   0.873   0.002   0.816   0.182
B A  2JELP                 10    0.157  17.290   0.410   0.020   0.205   0.775
E P  2JELP                 11    0.692  98.209   0.379   0.018   0.019   0.964
E N  2JELP                 12    0.430  62.923  -0.619   0.058   0.017   0.925
E G  2JELP                 13    0.274  21.603  -1.054   0.053   0.043   0.903
B L  2JELP                 14    0.096  17.523   0.224   0.113   0.043   0.844
B H  2JELP                 15    0.160  29.159  -0.638   0.191   0.086   0.723
B T  2JELP                 16    0.055   7.573  -0.196   0.354   0.048   0.598
B R  2JELP                 17    0.238  54.525   1.161   0.522   0.016   0.462
B P  2JELP                 18    0.065   9.223   0.299   0.858   0.002   0.139
B A  2JELP                 19    0.027   3.019  -0.111   0.970   0.001   0.030
B A  2JELP                 20    0.129  14.205  -0.747   0.970   0.001   0.030
E Q  2JELP                 21    0.318  56.813   2.073   0.970   0.001   0.030
B F  2JELP                 22    0.038   7.687   0.289   0.988   0.000   0.012
B V  2JELP                 23    0.086  13.172   0.982   0.988   0.000   0.012
E K  2JELP                 24    0.503 103.488   1.571   0.970   0.001   0.030
B E  2JELP                 25    0.192  33.455   1.405   0.970   0.001   0.030
B A  2JELP                 26    0.038   4.221  -0.767   0.923   0.002   0.076
E K  2JELP                 27    0.590 121.301   0.939   0.782   0.003   0.216
E G  2JELP                 28    0.628  49.424   1.346   0.430   0.016   0.555
B F  2JELP                 29    0.131  26.332   0.571   0.053   0.043   0.903
E T  2JELP                 30    0.701  97.284   1.489   0.004   0.085   0.910
B S  2JELP                 31    0.054   6.294  -0.501   0.005   0.336   0.660
E E  2JELP                 32    0.467  81.515   2.044   0.001   0.900   0.099
B I  2JELP                 33    0.028   5.106   0.783   0.000   0.983   0.017
E T  2JELP                 34    0.291  40.403   2.040   0.000   0.983   0.017
B V  2JELP                 35    0.029   4.380   0.657   0.000   0.983   0.017
E T  2JELP                 36    0.310  43.052   1.379   0.001   0.959   0.040
E S  2JELP                 37    0.344  40.352   0.888   0.003   0.718   0.279
E N  2JELP                 38    0.703 102.905   0.337   0.005   0.045   0.951
E G  2JELP                 39    0.828  65.171  -0.207   0.003   0.003   0.994
E K  2JELP                 40    0.557 114.637   1.228   0.005   0.336   0.660
E S  2JELP                 41    0.570  66.781   1.120   0.004   0.616   0.381
B A  2JELP                 42    0.055   6.017  -0.149   0.004   0.514   0.481
E S  2JELP                 43    0.380  44.489   1.068   0.004   0.138   0.858
B A  2JELP                 44    0.076   8.364  -1.508   0.600   0.003   0.397
E K  2JELP                 45    0.430  88.451   0.920   0.694   0.003   0.303
B S  2JELP                 46    0.165  19.385  -0.085   0.802   0.014   0.185
B L  2JELP                 47    0.062  11.352  -0.001   0.923   0.002   0.076
B F  2JELP                 48    0.125  24.987  -0.088   0.970   0.001   0.030
B K  2JELP                 49    0.183  37.623  -0.272   0.970   0.001   0.030
B L  2JELP                 50    0.054   9.832   0.067   0.970   0.001   0.030
B Q  2JELP                 51    0.084  15.038  -0.426   0.970   0.001   0.030
B T  2JELP                 52    0.197  27.324  -0.998   0.923   0.002   0.076
B L  2JELP                 53    0.065  11.902  -0.346   0.858   0.002   0.139
B G  2JELP                 54    0.113   8.925  -1.600   0.694   0.003   0.303
B L  2JELP                 55    0.066  12.030  -0.019   0.246   0.004   0.750
E T  2JELP                 56    0.454  62.956   0.695   0.115   0.016   0.868
E Q  2JELP                 57    0.525  93.819   0.745   0.018   0.019   0.964
E G  2JELP                 58    0.576  45.347  -0.962   0.005   0.015   0.979
B T  2JELP                 59    0.169  23.413   0.110   0.004   0.085   0.910
E V  2JELP                 60    0.497  76.404   1.944   0.001   0.900   0.099
B V  2JELP                 61    0.030   4.611   1.008   0.001   0.959   0.040
E T  2JELP                 62    0.296  41.111   2.346   0.000   0.983   0.017
B I  2JELP                 63    0.025   4.680   1.214   0.000   0.983   0.017
E S  2JELP                 64    0.307  35.992   1.838   0.001   0.959   0.040
B A  2JELP                 65    0.030   3.306  -0.031   0.001   0.900   0.099
E E  2JELP                 66    0.524  91.543   1.140   0.022   0.411   0.567
B G  2JELP                 67    0.229  18.030  -0.287   0.005   0.045   0.951
E E  2JELP                 68    0.791 138.275   1.317   0.176   0.004   0.820
E D  2JELP                 69    0.402  57.971   1.032   0.246   0.004   0.750
B E  2JELP                 70    0.062  10.796  -0.109   0.923   0.002   0.076
E Q  2JELP                 71    0.585 104.535   1.788   0.970   0.001   0.030
E K  2JELP                 72    0.510 104.866   2.154   0.970   0.001   0.030
B A  2JELP                 73    0.029   3.141   0.905   0.970   0.001   0.030
B V  2JELP                 74    0.085  13.049   1.199   0.970   0.001   0.030
E E  2JELP                 75    0.590 103.090   2.164   0.970   0.001   0.030
E H  2JELP                 76    0.278  50.550   2.368   0.970   0.001   0.030
B L  2JELP                 77    0.026   4.669   1.176   0.988   0.000   0.012
B V  2JELP                 78    0.236  36.335   1.734   0.988   0.000   0.012
E K  2JELP                 79    0.548 112.621   2.117   0.970   0.001   0.030
B L  2JELP                 80    0.099  18.200   1.340   0.970   0.001   0.030
B M  2JELP                 81    0.077  15.488  -0.272   0.923   0.002   0.076
E A  2JELP                 82    0.597  65.767   1.830   0.858   0.002   0.139
E E  2JELP                 83    0.630 110.044   1.097   0.600   0.003   0.397
E L  2JELP                 84    0.363  66.484   0.711   0.406   0.004   0.590
E E  2JELP                 85    0.916 160.008   1.410   0.003   0.003   0.994

Explain the output.  Go back.
```

---
